# Sarcocystosis in South American camelids: The state of play revisited

**DOI:** 10.1186/s13071-018-2748-1

**Published:** 2018-03-06

**Authors:** Muhammad A. Saeed, Mohammed H. Rashid, Jane Vaughan, Abdul Jabbar

**Affiliations:** 1 Department of Veterinary Biosciences, Faculty of Veterinary and Agricultural Sciences, The University of Melbourne, Werribee, Victoria 3030 Australia; 2 Cria Genesis, PO Box 406, Ocean Grove, Victoria 3226 Australia

**Keywords:** Sarcocystosis, *Sarcocystis*, Alpaca, Llama, Guanaco, Vicuna

## Abstract

Members of the genus *Sarcocystis* (Apicomplexa: Sarcocystidae) are intracellular protozoan parasites that infect a wide range of domestic and wild animals, resulting in economic losses in production animals worldwide. *Sarcocystis* spp. have indirect life-cycles where canids and felids serve as main definitive hosts while a range of domestic and wild animals serve as intermediate hosts, including South American camelids (SACs) such as alpacas, llamas and guanacos. These animals primarily occur in South American countries on Andean, elevated plains but in recent years, alpacas and llamas have become emerging animal industries in other parts of the world such as Australia, Europe and the USA due to their high-quality fiber, meat and hides. For instance, alpaca meat is becoming popular in many parts of the world due to its lower cholesterol content than other red meat, thereby it has the potential of a valuable product for both local and international markets. However, SAC meat can be degraded and/or even condemned due to the presence of macroscopic sarcocysts in skeletal muscles, leading to significant economic losses to farmers. The infection is generally asymptomatic, though highly pathogenic or even fatal *Sarcocystis* infections have also been reported in alpacas and llamas. Despite the economic importance of sarcocystosis in SACs, little is known about the life-cycle of parasites involved, disease transmission, epidemiology, pathogenesis, diagnosis, control and public health significance. This review article provides an in-depth analysis of the existing knowledge on the taxonomy, epidemiology, clinicopathology and diagnosis of *Sarcocystis* in SACs, highlights knowledge gaps and proposes future areas of research that could contribute to our better understanding of sarcocystosis in these animals.

## Background

Sarcocystosis is a parasitic disease caused by intracellular protozoan parasites belonging to the genus, *Sarcocystis* (Apicomplexa: Sarcocystidae). There are more than 200 species of *Sarcocystis* recognized [1] that infect a wide array of domestic and wild animals worldwide, causing significant health and economic losses [1, 2]. Members of the genus *Sarcocystis* have indirect life-cycles where definitive hosts such as carnivores or omnivores (e.g. humans), reptiles and raptorial birds become infected following ingestion of infective stages of parasites and then following sexual development, excrete oocysts/sporocysts into the environment that could infect intermediate hosts. In intermediate hosts, including herbivorous animals, humans, nonhuman primates, birds, reptiles and carnivores, parasites enter endothelial cells where they undergo multiple generations of merogony (asexual development) and sarcocysts (microscopic or macroscopic depending on the species involved) develop primarily in skeletal muscles of the tongue, neck, diaphragm, and legs and cardiac muscles [1, 3, 4], though they have also been found in smooth muscles of the intestine and central nervous system [1, 5].

South American camelids (SACs), include alpacas (*Vicugna pacos*), llamas (*Lama glama*), vicunas (*Vicugna vicugna*) and guanacos (*Lama guanicoe*) and make up a total population of at least seven million. They are widely distributed in South America, with alpacas, llamas and vicunas ranging from southern Ecuador to northern Chile with high densities in Bolivia and Peru, and guanacos are found across southern Argentina and Chile [6–8]. Alpacas and llamas are domesticated farm animals while guanacos and vicunas are wild species. The greatest numbers of SACs are raised by Andean families, playing a crucial role in their socioeconomic status as these animals are a good source of meat, hides and quality fiber [7–9]. Given that SACs possess physiological adaptations to high altitude and arid conditions, they are suitable for commercial livestock farming not only at high altitudes with freezing temperatures but also in regions of low rainfall - a distinguishing feature from other domestic livestock [6, 9]. The consumption of alpaca meat is increasing in developed countries as it possesses lower fat and cholesterol than beef, sheep and goat meat which potentially makes it a highly valuable product for both local and international markets [10]. Due to their superior quality wool/fiber and meat, alpaca and llamas farming have become an emerging animal industry in many parts of the world, including Australia, Europe and the USA. For instance, Australia has one of the largest alpaca breeding herds in the world and also has the largest alpaca population (i.e. > 300,000) outside South America [9, 11]. However, SAC meat can be downgraded or condemned due to sarcocystosis and associated public health concerns [8, 12].

South American camelids serve as important intermediate hosts for *Sarcocystis* spp., with macroscopic sarcocysts (2–7 mm) appearing in various skeletal muscles of llamas and alpacas, thus rendering meat unfit for human consumption [1, 13, 14], and leading to economic losses [6, 15, 16]. *Sarcocystis* spp. usually cause subclinical infections in SACs, although fatal cases have also been reported [17, 18]. Sarcocystosis is an emerging disease in SACs [19], however little is known about its pathology, transmission, life-cycle, economic importance and public health significance.

This article is aimed to provide a systematic overview of the current knowledge on the pathogenesis, epidemiology, taxonomy and diagnosis of *Sarcocystis* spp. infecting SACs. Furthermore, it highlights future areas of research that could contribute to our better understanding of sarcocystosis in alpacas and llamas.

## Taxonomy of *Sarcocystis* spp. in South American camelids

Although both macroscopic (macrocysts) and microscopic (microcysts) sarcocysts have been reported in SACs (Table 1) [13, 14, 17, 18], it remained elusive until recently whether the two types of sarcocysts in SACs were caused by the same or different species of *Sarcocystis*. Thus, various names have been proposed to describe sarcocysts in SACs, resulting in considerable confusion regarding the nomenclature of *Sarcocystis* spp. infecting alpacas, guanacos and llamas.

**Table 1:** Morphology of *Sarcocystis* spp. in South American camelids

Species of Sarcocystis^a^	Type of tissue cyst	Host	Size of tissue cyst (mm)	Bradyzoite/ merozoite (μm)	Reference
*S. aucheniae*	Macroscopic	Llama	4–5	na	[38]
		Alpaca, llama, guanaco	2–7	13–18 × 3–5	[14]
		Guanaco	2–7	13–18 × 3–5	[13]
		Llama	na	17.7 × 3.6	[16]
		Alpaca	8 × 3	na	[18]
*Sarcocystis* sp.		Alpaca	0.4–0.8	2–4	[17]
*S. masoni*	Microscopic	Alpaca, llama, guanaco	0.8 × 0.035–0.095	11–14 × 2–3.5	[14]

*Abbreviation*: *na* not available

^a^The name used by the authors

The first macroscopic sarcocyst in SACs was reported from a llama about a century ago and the parasite was named as *Sarcocystis aucheniae*, though no further details were provided [20]. Decades later, sarcocysts were observed in a guanaco and the parasite was named as *S. tilopodi*, based on its presence in a different camelid host rather than morphological differences [21]. Gorman et al. [22] experimentally infected dogs and cats with macroscopic sarcocysts from guanacos and found that only dogs excreted parasites (sporocysts) in their feces. These authors proposed that the parasites should be called as *S. guanicoecanis* and *S. lamacanis* in guanacos and llamas, respectively; however, no morphological differences to differentiate these sarcocysts were described. Subsequently, it was proposed that macroscopic and microscopic sarcocyst-forming species of *Sarcocystis* in alpacas and llamas should be called *S. aucheniae* and *S. lamacenis*, respectively, although no explanation for this proposal was provided [6].

Until recently, various names, including *S. aucheniae*, *S. lamacanis* and *S. lamacenis* had been used in different studies [15, 23, 24]. However, recently Dubey et al. [1] proposed that *S. aucheniae* was the only valid name (as per standard nomenclature) for the *Sarcocystis* species that forms macroscopic sarcocysts in llamas and alpacas. Recently, microscopic cysts from alpaca, llama, and guanacos from South American countries were described and the parasite was named as *S. masoni* [14]. Hence, based on the current morphological and molecular evidence, only two valid species of *Sarcocystis*, *S. aucheniae* and *S. masoni*, infect SACs that form macroscopic and microscopic sarcocysts, respectively. However, in this review, we have used the original names of *Sarcocystis* spp. as used by various authors (Tables 2 and 3) as it was not possible for us to decide which type of sarcocyst was *S. aucheniae* or *S. masoni*.

**Table 2:** Studies aimed at assessing the prevalence and epidemiology of *Sarcocystis* spp. using different diagnostic methods in South American camelids

Species of Sarcocystis^a^	Host	Geographical location	Tissue examined	Method used	Percent prevalence (proportion)	Reference
*S. aucheniae*	Llama	Argentina	Serum	ELISA	36 (183/507)	[8]
	Llama	Argentina	Blood	PCR	na	[38]
	Alpaca, llama, guanaco	Argentina, Peru	Skeletal muscle	Ge, Mic, TEM, PCR	na	[14]
	Guanaco	Argentina	Skeletal muscle	Ge, TEM, PCR	100 (2/2)	[13]
	Llama	Argentina	Serum	Ge, PCR	100 (3/3)	[16]
	Llama	Bolivia	Skeletal muscle	Ge	35 (138/378)	[12]
			Serum	ELISA	45 (171/378)	
	Alpaca	Australia	Skeletal muscle	PCR	100 (1/1)	[27]
	Alpaca	USA	Skeletal muscle	Ge, His, TEM	100 (1/1)	[18]
	Llama	Bolivia	Skeletal muscle	Ge, Mic, TEM	na	[26]
*S. masoni*	Alpaca, llama, guanaco	Argentina, Peru	Skeletal muscle	Ge, Mic, TEM, PCR	na	[14]
*Sarcocystis* sp.	Guanaco	Chile	Skeletal muscle	Ge	37 (33/89)	[32]
	Llama	Chile	Skeletal muscle	Ge	100 (28/28)	
	Alpaca	Uruguay	Skeletal muscle	Ge	(4/na)	
	Llama	Bolivia	Skeletal muscle	Ge	34 (na/1196)	[15]
	Alpaca	Australia	Skeletal muscle	Ge, His, TEM	100 (1/1)	[17]
	Llama	Argentina	Serum	IFAT	96 (295/308)	[37]
	Alpaca	Peru	Serum	ELISA	90 (844/941)	[34]
	Guanaco	Argentina	Heart, skeletal muscle, tongue	Ge	67 (8/12)	[56]

*Abbreviations*: *G* gross examination, *His* histology, *IFAT* indirect fluorescent antibody test, *Mac* macroscopic, *Mic*, microscopic/microscopy, *na* not available/applicable, *TEM* transmission electron microscopy

^a^The name used by the authors

**Table 3:** Experimental studies on *Sarcocystis* spp. infecting South American camelids

Species of *Sarcocystis*^a^	Geographical location	Experimental animal	No. of animals infected	Infective tissue/material used	Infective dose	Reference
*S. aucheniae*	Peru	Dog	18	Alpaca meat	100 Mac	[45]
	Peru	Dog	5	Alpaca meat	400 Mac	[30]
	Peru	Dog	18	Alpaca meat	180–200 Mac	[47]
	Peru	Dog	13	Llama meat	200 g meat	[46]
	Peru	Dog	12	Llama meat	150–200 Mac	[43]
	Peru	Dog	26	Alpaca, llama meat	500 Mac	[31]
	Bolivia	Dog, cat	1, 1	Llama meat		[26]
*S. lamacanis*	Peru	Alpaca	63	Sporocyst	1 × 10^3^, 2.5 × 10^3^, 5 × 10^3^	[23]
	Peru	Alpaca	7	Sporocyst	3 × 10^4^	[24]
*S. guanicoecanis*	Chile	Dog, cat	4, 4	Guanaco meat	250 g, 50 g meat	[22]

*Abbreviation*: *Mac* macrocysts

^a^The name used by the authors

## Structure of sarcocysts found in SACs

**Fig. 1:**
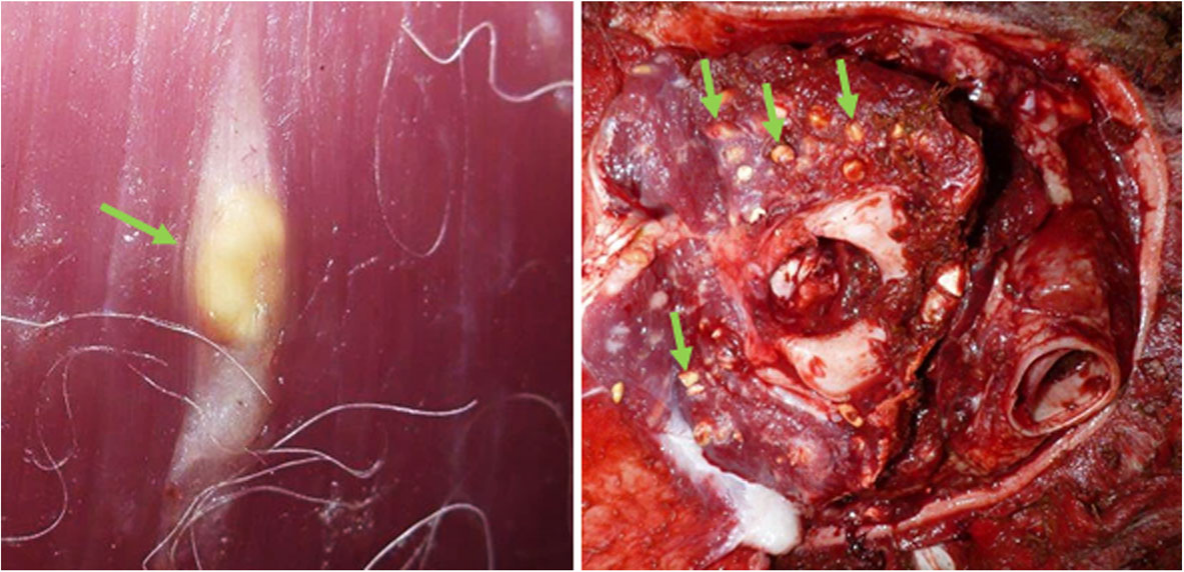
Macroscopic sarcocysts (arrows) in skeletal muscles of alpaca

Both macroscopic and microscopic sarcocysts have been reported from SACs [13, 14, 17, 18]. Grossly, macroscopic sarcocysts appear as rice-sized and shaped cysts in various skeletal muscles (Fig. 1) of alpacas, guanacos and llamas while microscopic sarcocysts are commonly found in cardiac muscles of alpacas and llamas [13, 14]. Generally, a sarcocyst consists of a primary cyst wall which contains numerous villar protrusions. The ground substance is found at the base of villar protrusions which extends inside the cyst cavity to divide it into numerous compartments, containing metrocytes and merozoites/bradyzoites [1]. The structure of primary cyst wall is considered the most important criterion to classify *Sarcocystis* spp. [1, 25].

### Macroscopic sarcocysts (*S. aucheniae*)

Macroscopic sarcocysts are 2–7 mm long, pale yellow in color and are surrounded by a dense (50 μm thick) secondary cyst wall. Schnieder et al. [26] described the first ultrastructure of macroscopic sarcocysts (*S. aucheniae*) isolated from llamas in Bolivia. Subsequently, several studies have described the ultrastructure of *S. aucheniae* macrocysts isolated from alpacas, llamas and guanacos from various countries [13, 14].

The cyst wall of a macroscopic sarcocyst can measure up to 10 μm thick (including the ground substance layer) and is folded into surrounding muscle fibers to form cauliflower-like villar protrusions. Each villar protrusion contains numerous microfilaments or microtubules [26]. The villar protrusions measure approximately 3.0–4.5 × 2.5–3.5 μm in size and some of them harbor a conical cap. Each sarcocyst contains 10–15 million bradyzoites (13–18 × 3–5 μm) which are oval to elongated in shape and packed in sacs separated by septa. Each bradyzoite contains several micronemes (secretory organelles) and amylopectin granules, clustered in anterior and posterior halves, respectively [13, 14]. Recent studies revealed that *18S* rRNA gene sequences of macroscopic sarcocysts had a sequence homology of 98–99% with those of previously published sequences of *S. aucheniae* from SACs [13, 14, 16, 27].

### Microscopic sarcocysts (*S. masoni*)

In 2016, More et al. [14] described the first ultrastructure of a microscopic sarcocyst (*S. masoni*) found in SACs. Microscopic sarcocysts can measure up to 800 μm in length and 35–95 μm in width, with a wall thickness of 2.5–3.5 μm. The cyst wall harbors conical to cylindrical villar protrusions with several microtubules and each villar protrusion consists of 11 or more rows of knob-like projections. Bradyzoites measure 11–14 × 2,0–3.5 μm in size and contain numerous micronemes and amylopectin granules. Molecular studies targeting full-length *18S* RNA gene sequencing revealed that this parasite had 95–96% identity with those of other *Sarcocystis* spp. available in the GenBank database [14].

## Life-cycle of *Sarcocystis* spp.

**Fig. 2:**
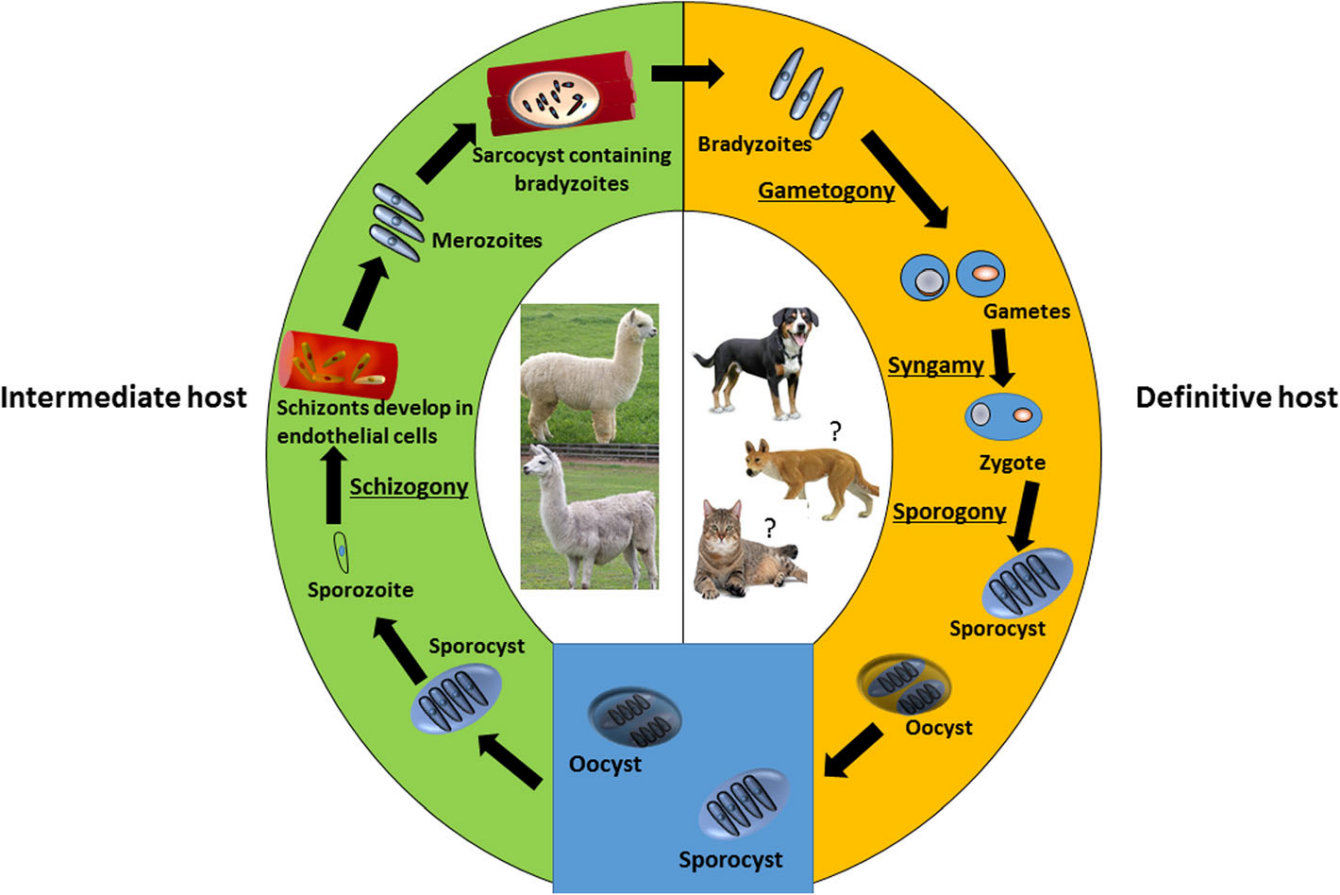
Life-cycle of *Sarcocystis* spp. in South American camelids

Although the precise life-cycle of *Sarcocystis* spp. in SACs is not completely known, a general life-cycle of a *Sarcocystis* spp. is given in Fig. 2. A camelid (intermediate) host becomes infected with *Sarcocystis* by ingesting sporulated oocysts or sporocysts from the environment [25, 28]. Each sporocyst contains four sporozoites which are liberated in the digestive tract (following exposure to trypsin or bile) and move to penetrate endothelial cells of blood vessels where they undergo asexual multiplications (schizogony) [1, 29]. Schizonts of *Sarcocystis* spp. multiply by endopolygeny which results in budding of merozoites at the surface of the schizont. Following a few generations of schizogony (depending on *Sarcocystis* spp.), a large number of merozoites are produced which migrate to muscles through blood [1]. Merozoites develop into sarcocysts inside parasitophorous vacuoles (made from host cell plasma membrane to protect sarcocysts from host cell defense mechanisms) in myocytes which contain millions of infective bradyzoites [1, 25]. A major structural difference between merozoites and bradyzoites of all *Sarcocystis* spp. is the absence of rhoptries in merozoites [1]. The number of generations (cycles) of *Sarcocystis* schizonts (schizogony) and their exact location in the camelid host remains elusive. However, four generation of schizonts have been reported for *S. cruzi* which first appear within endothelial cells of small arteries while subsequent generations are found in arterioles, and then capillaries and veins in various parts of the body until the last generation which develops in muscles [1].

A canid (definitive) host acquires infection following the ingestion of infected meat containing sarcocysts. Bradyzoites are released from the cyst and invade intestinal epithelial cells and undergo gametogony (sexual reproduction) which results in the formation of macrogametes and microgametes. Finally, macro- and micro-gametes fuse to form a zygote in the lamina propria of the small intestine of the definitive host and develop into the oocyst. Oocysts have delicate membrane, which frequently ruptures, releasing sporocysts (which thus can be free, or still encapsulated) in feces and may contaminate food and water consumed by intermediate hosts (e.g. SACs) [1, 25, 29].

A number of studies have been conducted to establish the definitive host(s) of *Sarcocystis* spp. that infect SACs (Table 3). Various potential definitive hosts, including dogs and cats, were fed with alpaca, llama and/or guanaco meat containing macroscopic sarcocysts, and following a period of time, feces were examined for the presence of sporocysts [22, 26]. To date, only dogs (fed with infected camelid meat) have been found to produce sporocysts (14.6–15.0 × 10.4–10.6 μm) in their feces, with variable prepatent (9–16 days) and patent (19–61 days) periods [22, 26, 30, 31]. These studies indicate that the dog is the potential definitive host for *S. aucheniae*, the macroscopic cyst forming species. However, the sporocyst burden and prepatent periods in dogs could vary depending on the size of the macrocyst they receive. For instance, the puppies that received smaller size (1–3 mm) macrocysts shed significantly more sporocysts with a shorter prepatent period (12 days), compared to those (16 days prepatent period) which received larger (˃ 5 mm) macrocysts [31]. The definitive host for *S. masoni*, the microcyst forming species, remains unknown although the dog and other canids have been suggested as the potential definitive host(s) for this parasite [14].

## Epidemiology of sarcocystosis in SACs

Very few studies have been undertaken to understand the epidemiology of sarcocystosis in SACs. Table 2 summarizes key studies on the prevalence of *Sarcocystis* spp. in these animals. Natural infections with *Sarcocystis* spp. in SACs are mostly asymptomatic [8, 15, 32]; however, significant pathology could be observed in affected tissues [17]. The prevalence of *Sarcocystis* infections in SACs has been estimated, including alpacas (90–100%), llamas (34–100%) and guanacos (37–100%) (Table 2). However, caution must be taken when comparing these prevalence figures due to differences in the sampling frame, diagnostic methods used, animal age, type of tissue(s) analyzed and the number of samples used in these studies. To date, no epidemiological information is available for sarcocystosis from vicunas.

Poor sanitation and the presence of pastoral dogs are considered as risk factors for sarcocystosis in SACs. For instance, a higher prevalence (50%) of sarcocystosis was observed in llamas kept under poor sanitary conditions and in the presence of pastoral dogs, compared to those (23 and 26%) kept at different localities but under good sanitary conditions and in the absence of pastoral dogs [8]. These results indicate that herd management practices could greatly impact the exposure of camelids to *Sarcocystis*. In fact, infective sporocysts of *Sarcocystis* spp. are passed in faeces of the definitive host and they could remain infective for many months. Furthermore, their maturation and infectivity are not dependent on weather conditions unlike many other coccidian parasites [33] which could explain high infection rates in llamas kept under poor sanitary conditions [8].

Location and feeding practices have been found to affect the prevalence of sarcocytosis in SACs. For example, the prevalence of macroscopic sarcocysts in commercially slaughtered llamas from Bolivia varied between 23% (2007) and 50% (2011) with an overall prevalence of 34% [15]. In another study, a high prevalence (100%) of *Sarcocystis* was observed in llamas slaughtered in Chile [32] (Table 2). This difference in the infection rates could be due to variation in geoclimatic location and potentially feeding practices as a higher prevalence of *Sarcocystis* was reported in guanacos grazing on pastures than those from forested areas [32]. The high prevalence in the former group was possibly due to frequent contact of these animals with dogs used for shepherding - the potential definitive hosts for *Sarcocystis* [32]. However, it has been demonstrated recently in Argentina that sarcocystosis in llamas remained unaffected by climate, altitude or pasture characteristics [8] which warrant for further studies.

Age has been shown to be a risk factor for *Sarcocystis* infections in llamas and alpacas as the older animals harbor sarcocysts more frequently than the younger ones [8, 15, 34] as they have been exposed to potentially infected pasture for longer. Likewise, females and long-haired llamas had higher infection rates than males and short/intermediate-haired llamas, respectively, indicating that gender and breed could also contribute to developing sarcocysts in SACs [15]. Gestation and parturition, especially the first parturition may negatively impact immunity in female camelids which could contribute to developing sarcocysts in these animals. However, in a recent study male gender was regarded as a risk factor for developing sarcocystosis in llamas [8] which requires further investigation.

SACs are most likely infected by ingestion of food and water contaminated with sporocysts from dog feces. Thus, disrupting the *Sarcocystis* life-cycle between SACs and the potential definitive hosts such as dogs could be an effective strategy for controlling sarcocystosis in SACs. The lactogenic transfer of *Sarcocystis via* milk or colostrum could be another potential method of transmission in SACs though experimental infection studies failed to transfer *Sarcocystis* spp. from cows to calves via these methods [1]. Transport hosts (e.g. birds) have been reported to disseminate sporocysts for some *Sarcocystis* spp. [35], although the role of transport host remains to be established for *Sarcocystis* spp. infecting SACs.

## Pathogenesis of sarcocystosis in SACs

Very little is known about the pathogenesis of sarcocystosis in SACs. Generally, the number and distribution of sarcocysts in intermediate hosts depend on several factors, including the number of sporocysts ingested, the immune status of the host and *Sarcocystis* spp. involved [1, 28, 36]. However, none of these factors have yet been explored fully for sarcocystosis in SACs.

Sarcocystosis is usually asymptomatic in SACs, though multiple superficial abscesses in the neck have been observed occasionally. Fatal clinical cases ascribed to macroscopic sarcocystosis have also been reported. For instance, eosinophilic myositis was reported in the USA in an alpaca naturally infected with *Sarcocystis* [18] and it exhibited clinical signs, including recumbency, hypothermia and non-responsiveness to external stimuli, dyspnea, marked muscle tremors, reduced milk production and abortion. At post-mortem, white macroscopic sarcocysts (8 × 3 mm) in skeletal muscles and hemorrhages in myofibers were observed throughout the carcass [18]. A similar case of necrotizing and histiocytic myositis was reported from an alpaca in Australia [17]. The aspirate analysis of subcutaneous abscess-like structures along the head and cervical region revealed the presence of large numbers of eosinophils. Examination of hematological parameters showed peripheral eosinophilia, hyperproteinemia and hyperglobulinemia. At post-mortem, multiple foci of caseating, pale, white, streaking lesions were noted on the cranial and cervical musculature. Histologically, the infected tissues were characterized by multifocal caseous necrosis associated with histiocytes, giant cells and lymphocytes [17]. A significant destruction and necrosis in tissues could be explained by rapid multiplication of asexual developmental stages, though it requires further investigation. However, localized tissue necrosis alone may not be enough to cause severe illness or death seen in large animals [1]. Overall these studies indicate that *Sarcocystis* could be highly pathogenic or even fatal in SACs.

Recently, the effect of microscopic sarcocysts was studied in young alpacas, experimentally infected with sporocysts [23]. The infected alpacas showed decreased body weight gain and hematocrit levels compared to control groups. A high mortality (92%) was observed in alpacas infected with a high dose of sporocysts [23]. Histological examination of cardiac muscle revealed that microscopic sarcocysts were located only in myocytes and not in Purkinje cells and they did not cause any interruption in the conduction of electrical impulses through the myocardium [24].

Overall these studies indicate that *Sarcocystis* spp. can account for significant pathology in SACs, although the infection usually remains subclinical or asymptomatic. Further studies are required to understand the pathogenesis of both macro- and microscopic sarcocystosis and their impact on musculoskeletal and cardiac function, immunity and productivity of SACs, especially in terms of animal welfare and economic importance.

## Diagnosis of sarcocystosis in SACs

There are no standard criteria or commercial tests available for the diagnosis of sarcocystosis in SACs. The identification of sarcocysts in muscles of alpacas and llamas at necropsy has been used as the sole definitive diagnostic method in most of the studies (Table 2). The disease can be diagnosed based on the elimination of other parasites with similar clinicopathology and an evaluation of epidemiological information. However, the diagnosis of acute sarcocystosis is challenging as the disease may be asymptomatic or generalized in nature, with no specific signs and it is unlikely that sarcocysts would be detected in tissues at this stage.

A few studies have utilized serological methods for the diagnosis of sarcocystosis in SACs. For instance, a serological test was developed to diagnose anti-*Sarcocystis* antibodies in llama serum, where soluble antigen was isolated from macrocysts collected from alpacas naturally infected with *S. aucheniae* [12]. However, due to its low sensitivity and specificity (~65% for each), it was concluded that the test was unsuitable for the detection of sarcocystosis in individual animals [12]. Subsequently, an indirect fluorescent antibody test (IFAT) was developed to determine the seroprevalence of sarcocystosis in llamas [37]. Using bradyzoite-derived antigens from *S. aucheniae* as well as *S. cruzi* (cause sarcocystosis in cattle), the test allowed the detection of anti-*Sarcocystis* antibodies in 96% of llama serum tested. However, cross-reactivity was also observed between *Sarcocystis* spp. since two different types of bradyzoites (*S. aucheniae* from llama and *S. cruzi* from cattle) were used [37], thereby making this test unsuitable for species identification. Recently, Romero et al. [8] developed an indirect enzyme-linked immunosorbent assay (ELISA) to detect anti-*Sarcocystis* antibodies in serum from llamas. They used the *Sa23* protein antigen which is a 23 kDa soluble immunogenic fraction of *S. aucheniae* macrocysts. Hence, these studies indicate that serological diagnosis of sarcocystosis in SACs is possible; however, none of the studies defined the precise nature of antigens used or verified serological diagnosis with molecular or histopathological diagnoses.

A variety of molecular methods have been developed for assessing the genetic diversity as well as for the diagnosis of *Sarcocystis* spp. in animals [16, 38, 39]. The first molecular identification of macrocysts of *S. aucheniae* in alpacas was made in Australia where the *18S* rRNA gene fragment was amplified using conventional polymerase chain reaction (PCR) [27], and the phylogenetic analyses of the *18S* rRNA sequences revealed that these were different from those of *Sarcocystis* spp. that infect other ruminants. Subsequent studies also amplified *18S* rRNA gene from macrocysts of *S. aucheniae* collected from llamas [16] and guanacos [13], and these *18S* rRNA sequences from different SAC species were homologous, suggesting that *S. aucheniae* infects three species (i.e. alpacas, llamas and guanacos) of SACs and can potentially be diagnosed using common molecular method(s). Molecular diagnosis from sarcocysts is invaluable in identifying *Sarcocystis* at the species level; however, this method may have little value in the early diagnosis of sarcocystosis in SACs as the DNA is isolated from a developed cyst. Contrarily, the detection of *Sarcocystis* DNA from body fluids (such as blood) could be a much more valuable diagnostic tool for early diagnosis, particularly for the identification of microcyst-forming *Sarcocystis* spp. Recently, a semi-nested PCR was developed to detect the DNA of *S. aucheniae* from blood in llamas [38]. This method allowed the detection of as few as 100 bradyzoites per ml of blood. However, this test was not field-validated for the diagnosis of *Sarcocystis* spp. from the blood of naturally or experimentally infected SACs.

## Zoonosis and food safety with sarcocystosis

Currently known *Sarcocystis* spp. with zoonotic potential are *S. hominis*, *S. heydorni* and *S. suihominis*, all of which use humans as definitive host; the former two utilize cattle while *S. suihominis* uses pigs as intermediate hosts [29, 40]. *S. nesbitti* is another important *Sarcocystis* spp. which can infect humans (intermediate host) following the ingestion of food or water contaminated with reptile feces and snakes are considered as the potential definitive hosts for this parasite [41, 42]. It has been suggested that *Sarcocystis*-infected meat contains a cyst-derived neurotoxin called sarcocystin which can cause gastroenteritis, diarrhea, nausea, shivering and respiratory problems in human if the uncooked infected meat is consumed [6, 16, 29]. Although SAC meat infected with *Sarcocystis* spp. can cause significant pathology in dogs and cytotoxicity in rabbits, their zoonotic potential has not been established [6, 43, 44].

Physical methods of treating infected meat have been used to inactivate the protein toxin and the viability of sarcocysts. For example, llama meat naturally infected with *S. aucheniae* macrocysts was treated with different physical methods such as boiling (100 °C for 10 min), baking (105 °C for 65 min), freezing (-20 °C for 10 days) and frying, and then was fed to young dogs [45, 46]. The dogs that received treated meat did not pass any sporocysts in their feces whereas those who received untreated meat did pass sporocysts. In experimental rabbits, frozen meat consumption led to the development of moderate signs of toxicity (e.g. prostration, dyspnea, miosis, hyperthermia, diarrhea). Similarly, freezing (-18 °C to -24 °C) for five days or cooking (above 60 °C) was shown to be effective in inactivating sarcocysts in guanaco meat; however, refrigeration (for 30 days) was ineffective [22]. These results indicate that boiling, baking, frying and to some extent freezing can neutralize the viability and toxicity of macrocysts.

Chemical methods such as hot and cold smoking, dry curing and marination have also been used to detoxify llama and alpaca meat naturally infected with sarcocysts of *S. aucheniae* [43, 45]. All these methods were found effective in eliminating the viability of sarcocysts as dogs did not pass any sporocysts after receiving the chemically treated meat [43, 45]. However, variable toxicity results were observed in rabbits that received chemically treated meat [43, 45, 47]. This indicates that not all chemically-treated methods are effective in neutralizing the toxicity of sarcocysts.

## Control and prevention of sarcocystosis in SACs

There is no vaccine available to protect camelids or other domestic animals against sarcocystosis [1, 19, 29]. Immunization studies in domestic animals such as cattle, sheep, goats and pigs have shown that animals inoculated with *Sarcocystis* sporocysts were protected against a challenge dose of the parasite that normally would have been lethal [1, 48, 49]. This indicates that a vaccine could potentially be developed against *Sarcocystis* spp. of SACs and other domestic animals. However, a thorough understanding of immune responses of animals against *Sarcocystis* spp. is a pre-requisite for the vaccine development which has been a totally untouched area of research in SACs to date.

Anticoccidial drugs have been used to treat sarcocystosis in both definitive and intermediate hosts. For example, a severe form of sarcocystosis was prevented when camels experimentally infected with *Sarcocystis* sp. were treated with Amprolium® [50]. Other drugs such as salinomycin and halofuginone have also been used to reduce or prevent acute sarcocystosis in domestic animals infected with *Sarcocystis* spp. [1, 19, 51]. Notably, such drugs could be effective only in treating intestinal stages of *Sarcocystis* during the acute phase, which is almost impossible to detect under field conditions. Furthermore, such antiparasitic drugs not only could be toxic to SACs [52] but may have no value in treating sarcocysts developed in muscles. Therefore, prevention is the only practical solution to control sarcocystosis in SACs.

South American camelids develop sarcocystosis by ingesting sporocysts from a contaminated environment arising from infected faeces of carnivores. Although the life-cycle of *Sarcocystis* in SACs is not precisely known, the following steps could be followed to potentially disrupt the life-cycle and control of sarcocystosis in SACs: (i) domestic and wild carnivores should be excluded from animal housing and from feed, water, and bedding for SACs; (ii) farm dogs should be prevented from defecating on pastures and animal food/bedding storage sites; (iii) uncooked/untreated camelid meat should not be fed to dogs as it may contain sarcocysts; and (iv) alpaca and llama carcasses and camelid foetal/placental material should be kept away from dogs and wild carnivores by burying or incineration.

## Research gaps and future implications

Currently, the classification of *Sarcocystis* spp. infecting SACs is primarily based on the morphology of cyst wall of sarcocysts. For example, the cyst wall of *S. aucheniae* is characterized by cauliflower-like villar protrusions while that of *S. masoni* harbors conical to cylindrical villar protrusions. However, it is important to consider that physical features such as the type of cyst wall or cyst size may vary with the age/development of the cyst, location and type of the host cell, and tissue processing/fixation method, therefore caution must be taken while describing a *Sarcocystis* sp. based on morphological characters alone [1, 2, 36]. Furthermore, most of the studies describing the structure of sarcocysts have examined only a small number of cysts to describe new species of *Sarcocystis* in SACs [13, 14]. Thus, it is imperative to use molecular tools besides morphological characterization, with a large sample size for the robust and authentic taxonomy of *Sarcocystis* spp. that infect SACs.

To date, only dogs have been found to be a definitive host of *Sarcocystis* spp. that infect SACs. However, the role of wild/feral canids as definitive hosts of these species of *Sarcocystis* should be not be ignored as sarcocysts have been reported in other camelids (e.g. camels) in the absence of their known definitive host (dogs) [53]. Furthermore, the movement of SACs from their native South America to new geographical locations such as Australia and the USA, and the presence of *Sarcocystis* in camelids in these locations highlight the possibility of an involvement of new definitive host(s) such as dingoes (in Australia) under different climatic conditions. Future studies are required to establish the types of definitive host(s) of different species of *Sarcocystis* that infect SACs, thereby increasing our understanding about sarcocystosis in these animals and helping to design effective control strategies.

The lack of availability of a standard diagnostic test has impeded our ability to fully understand the epidemiology, clinical pathology and public health significance of sarcocystosis in SACs. Furthermore, the assessment of sensitivity and specificity of the new tests is challenging due to the lack of validated techniques for the diagnosis of sarcocystosis in SACs. A validated test that could allow the early detection of *Sarcocystis* from blood in SACs would be a benchmark for the diagnosis of sarcocystosis. Studies indicate that early diagnosis of sarcocystosis in SACs is feasible with serological methods, although a high inter-species cross-reactivity exists among *Sarcocystis* spp. infecting various ruminant species [8, 37]. Thus, it is important to consider the following factors for successful development and implementation of a serological test, including (i) the precise nature of antigens used; (ii) the type of antibodies (e.g. IgG, IgM) detected in serum; (iii) the correlation concordance between premortem serological and post-mortem histopathological diagnosis; and (iv) the sensitivity and specificity of the assay. Furthermore, a serological test for the diagnosis of sarcocystosis may require large numbers of highly purified parasites. The possible sources of purified parasites could be experimentally infected alpacas and llamas and *in vitro* cultivation of the parasite [54]. Molecular diagnosis of *Sarcocystis* spp. from blood could be another potential method for the early detection of *Sarcocystis* in SACs [38]. Studies have shown that merozoites could be detected in buffy coat preparations from cattle experimentally infected with *Sarcocystis* sp. as early as day 16 post-infection [55]. Although this method is tedious and not practical for routine diagnosis of sarcocystosis, it highlights the potential implication of PCR-based detection of *Sarcocystis* DNA from blood of SACs.

There is a paucity of information on the developmental stages (e.g. merozoites, macrogametes, microgametes etc.) of the parasite in both the camelid and definitive hosts. Although some asexual stages of *S. aucheniae* have been described *in vitro* [54], experimental infection studies are required to better understand the developmental biology and pathogenesis of *Sarcocystis* spp. in SACs as well as their definitive host(s).

## Conclusions

This article highlights the importance of sarcocystosis and comprehensively reviews the existing knowledge on sarcocystosis in SACs across the globe. SACs serve as important intermediate hosts for at least two morphologically and molecularly distinct *Sarcocystis* spp., *S. aucheniae* and *S. masoni*, that form macroscopic and microscopic sarcocysts, respectively. Macroscopic sarcocysts commonly occur in skeletal muscles while cardiac muscles appear to be the predilection site of microcysts. Generally, sarcocystosis remains asymptomatic, although fatal clinical cases have been reported in SACs. Age, poor sanitary conditions and the presence of dogs are some of the risk factors for the development of sarcocystosis in SACs. Several diagnostic tests have been used to diagnose sarcocystosis in SACs but a diagnostic method for an early detection of infection with *Sarcocystis* spp. is yet to be developed. Currently, no effective methods are available for the treatment and control of sarcocystosis in SACs. Although this article has provided a detailed analysis of the existing body of knowledge on sarcocystosis in SACs, a number of other aspects of this disease in alpacas and llamas still require further work to enhance our understanding about the impact of sarcocystosis on animal health in terms of productivity, weight gain, reproductive efficiency, wool production, immunity, musculoskeletal functioning, susceptibility to other diseases and economic losses.

## Abbreviations

ELISA: enzyme-linked immunosorbent assay
G: gross examination
His: histology
IFAT: indirect fluorescent antibody test
SAC: South American camelids
TEM: transmission electron microscope
